# JRL-YOLO: A Novel Jump-Join Repetitious Learning Structure for Real-Time Dangerous Object Detection

**DOI:** 10.1155/2021/5536152

**Published:** 2021-04-01

**Authors:** Yiliang Zeng, Lihao Zhang, Jiahong Zhao, Jinhui Lan, Biao Li

**Affiliations:** ^1^Department of Instrument Science and Technology, School of Automation and Electrical Engineering, University of Science and Technology Beijing, Beijing, China; ^2^Shunde Graduate School, University of Science and Technology Beijing, Beijing, Guangdong, China; ^3^Beijing Engineering Research Center of Industrial Spectrum Imaging, Beijing, China; ^4^School of Electrical, Computer and Telecommunications Engineering, University of Wollongong, Wollongong, New South Wales (NSW), Australia

## Abstract

Campus security incidents occur from time to time, which seriously affect the public security. In recent years, the rapid development of artificial intelligence has brought technical support for campus intelligent security. In order to quickly recognize and locate dangerous targets on campus, an improved YOLOv3-Tiny model is proposed for dangerous target detection. Since the biggest advantage of this model is that it can achieve higher precision with very fewer parameters than YOLOv3-Tiny, it is one of the Tinier-YOLO models. In this paper, the dangerous targets include dangerous objects and dangerous actions. The main contributions of this work include the following: firstly, the detection of dangerous objects and dangerous actions is integrated into one model, and the model can achieve higher accuracy with fewer parameters. Secondly, to solve the problem of insufficient YOLOv3-Tiny target detection, a jump-join repetitious learning (JRL) structure is proposed, combined with the spatial pyramid pooling (SPP), which serves as the new backbone network of YOLOv3-Tiny and can accelerate the speed of feature extraction while integrating features of different scales. Finally, the soft-NMS and DIoU-NMS algorithm are combined to effectively reduce the missing detection when two targets are too close. Experimental tests on self-made datasets of dangerous targets show that the average MAP value of the JRL-YOLO algorithm is 85.03%, which increases by 3.22 percent compared with YOLOv3-Tiny. On the VOC2007 dataset, the proposed method has a 9.29 percent increase in detection accuracy compared to that using YOLOv3-Tiny and a 2.38 percent increase compared to that employing YOLOv4-Tiny, respectively. These results all evidence the great improvement in detection accuracy brought by the proposed method. Moreover, when testing the dataset of dangerous targets, the model size of JRL-YOLO is 5.84 M, which is about one-fifth of the size of YOLOv3-Tiny (33.1 M) and one-third of the size of YOLOv4-Tiny (22.4 M), separately.

## 1. Introduction

In the past few years, there have been occasional high-profile and horrific acts of violence on campus [1]. Statistical analysis shows that violent incidents on campus are rare [2, 3]; although the incidence is extremely low, these incidents are indeed so terrible and painful that they seriously endanger campus security and social security. Therefore, it is essential to strengthen the management to maintain campus safety.

With the development of information technology, computer visual research has become a hotspot in the field of academic and industrial applications [4]. Especially after the introduction of artificial intelligence theory, more and more artificial intelligence products are brought into people's lives. Although there is a big gap between the visual understanding ability of machines and human beings, machines can be widely used in long-term and extremely harsh working environments [5]. Machines can also help us detect abnormal events in time and make early emergency response.

Traditional methods are used to identify specific human activities by detecting gestures, using online alarm system to detect abnormal situations in the campus environment [6, 7]. Although real-time monitoring is possible, traditional methods have limited detection features and cannot simultaneously and accurately distinguish various types of dangerous events. And Wu and Cheng [8] used traditional methods, but there are many parameters that need to be manually determined. The rapid development of computer technology provides a strong support for the development of object detection algorithms with high computational performance. Compared with traditional object detection algorithms, the deep learning algorithm has a great advantage in object detection.

Deep learning is now the mainstream method of dangerous object detection. Aiming at the shortcoming of existing feature classifiers with high false detection rate in target detection, a convolutional neural network detection algorithm based on deep learning was proposed to improve the robustness of the algorithm in complex environments [9]. A dangerous substance image detection method based on SSD was proposed in [10]. However, the detection effect of the model for large objects is very good, whereas the detection of small targets is not robust. YOLOv3 has been used to detect dangerous objects such as knives and guns in massive videos, which improves the detection accuracy of small targets, but the large model is difficult to be applied to small devices [11]. Hu et al. [12] had good effect on small targets, but it also cannot guarantee real-time performance.

In order to solve the problem of low efficiency of video anomaly detection, an adaptive key frame screening method based on the characteristics of abnormal targets was proposed in [13]. However, the application of this approach of abnormal behavior detection is limited, which is not conducive to its popularization. Xia et al. [14–17] all used deep learning to detect abnormal actions of the human body and achieved good results with strong robustness to noises in the environment. However, due to the large number of parameters in the above model, it does not consider the problem of real-time performance in small devices. Ji et al. [18] proposed an abnormal target detection algorithm based on the Tiny-YOLO network model. At the same time, the network is cut out and transplanted to the embedded hardware platform TX2. However, the final result cannot realize real-time detection. Fan et al. [19] used the spatiotemporal autoencoder and CNN for training, and real-time detection can be achieved at last, but the boundary of dangerous actions is not clearly defined, being prone to result in misidentifications.

In 2012, Krizhevsky et al. proposed the AlexNet method based on the convolutional neural network (CNN) [20], which proved that the features extracted by the CNN were more effective than traditional methods and led to higher classification accuracy. Girshick et al. [21] proposed R-CNN to significantly improve the detection performance by combining a large number of convolutional neural networks with the region proposal network (RPN). Fast R-CNN solves the problem of time-consuming and slowness of the traditional R-CNN [22]. Faster R-CNN introduces a fully convoluted RPN to share fully convoluted features, further reducing the amount of computation and improving the detection speed [23].

The detection of these two types of targets, dangerous objects and dangerous actions, is usually conducted by separate algorithms, and there is no integrated model yet. Although the deep learning algorithm has high detection accuracy, it has high requirements for hardware devices. In terms of speed, it is difficult to achieve real-time detection for limited hardware facilities such as mobile phones and embedded development boards. Therefore, it is necessary to explore a lightweight and high-precision object detection algorithm model.

YOLO (You Only Look Once) [24–27] is a one-stage object detection algorithm. The lightweight network YOLOv3-Tiny, which is a simple version of the YOLOv3 network, can achieve good results of open datasets and can detect targets in real time.

In this paper, a new YOLOV3-Tiny backbone network is proposed to design a more lightweight and efficient campus dangerous target detection model. The main work of this paper is summarized as follows:

1. Considering dangerous objects and dangerous actions, respectively, it is difficult to capture the characteristics of both “shape-unchanged appearance” and “shape-changeable appearance” at the same time. To redefine the campus of the dangerous objects and dangerous behaviors, the two types of targets are integrated into one model, which is lightweight and can achieve higher precision with fewer parameters.
2. In view of the missing detection phenomenon of objects in the practical application of YOLOv3-Tiny, a jump connection repeatedly learning structure is proposed, which is combined with the SPP structure, as a new backbone network of YOLOv3-Tiny. Its performance is improved compared to YOLOv3-Tiny, especially in the detection of finite-pixel targets.
3. Inspired by the DIoU-NMS algorithm, the soft-NMS algorithm is improved by adding the measurement of the center distance of the detection box, which has better performances than YOLOv3-Tiny's own NMS. It can effectively reduce the missing detection phenomenon when two targets are too close.

Experimental results show that the jump-join repetitious learning YOLO (JRL-YOLO) not only has high cost performance in practical applications but also can achieve higher precision with fewer parameters than YOLOv3-Tiny.

The rest of the paper is organized as follows. In Section 2, the main ideas of YOLOv3-Tiny are briefly reviewed.  Section 3 introduces our proposed JRL-YOLO, and Section 4 presents experimental results and analysis, with Section 5 concluding our work and giving advices for future work.

## 2. Brief Introduction of YOLOv3-Tiny

YOLOv3-Tiny [26] is a one-stage target detection algorithm, which transforms the target detection problem into a regression problem. It can directly obtain the target position information and category information without the need of the RPN, which is faster than Faster R-CNN. Compared with Faster R-CNN, real-time target detection can be achieved in a lower hardware device.

### 2.1. Principle of YOLO

YOLO will resize the image into a fixed size and feeds it to the network so that each detection layer has a fixed-size feature scale. YOLO uses the convolutional neural network to extract the depth features of the target and then divides the image into *S∗S* grids for grid-by-grid detection. It is assumed that there are *M* detection layers in YOLO, and each detection layer has *B* anchor boxes. Each detection layer should detect each grid and calculate the confidence category probability of each grid. The end result is that an object might have multiple bounding boxes; however, it is usually expected to see one object corresponding to only one bounding box in the end, so YOLOv3-Tiny uses the NMS algorithm to suppress the redundant bounding boxes.

### 2.2. Target Real-Time Detection Network of YOLOv3-Tiny

YOLOv3-Tiny is lighter than the YOLOv3 network structure and has a good effect on target detection. As shown in Table 1, the backbone network of YOLOv3-Tiny mainly consists of 7 convolutional layers of a 3 × 3 size and 6 pooling layers. The first five pooling layers have a stride size of 2, and the last one has a stride size of 1. YOLOv3-Tiny detection layers include shallow network detection and deep network detection. The detection of the shallow network is located after the backbone network, and the detection of the deep network is located behind the shallow network. The feature concat is obtained under different convolution kernel sizes in the shallow and deep layer so as to fuse the shallow and deep features to improve the accuracy of the model.

YOLOv3-Tiny is an end-to-end detection model whose input image is uniformly resized to a fixed size before entering the network. Generally, the input image is resized to a size of 608 × 608 or 416 × 416. YOLOv3-Tiny contains two YOLO layers to detect different scales, and the detection feature scale of the YOLO layer is related to the size of the input image. When the input image size is 416 × 416, the output feature sizes of the two YOLO layers are 13 × 13 and 26 × 26, respectively.

### 2.3. YOLOv3-Tiny Nonmaximal Suppression Algorithm

YOLOv3-Tiny's nonmaximal suppression (NMS) algorithm sorts all bounding boxes by the size of the score. First, the bounding box with the highest score is selected, and then a threshold is predetermined. When the intersection over union (IOU) of other bounding boxes and the bounding box of the highest score is larger than this threshold, the bounding box will be inhibited. This method can effectively reduce the number of overlapping boxes. However, when two objects are close to each other, the bounding boxes generated by the two objects are also close to each other. Similarly, the bounding boxes will inhibit each other, leading to one of the bounding boxes being eliminated. At this point, the phenomenon of missing detection of the object occurs.

## 3. Proposed Method

### 3.1. Proposed Structure of the Network

Previous methods, such as SSD and YOLOv3, regard target detection as a regression problem. Although they can achieve accurate and real-time detection effect, they are not good to run on embedded systems or other small-sized equipment. This paper introduces details of the proposed JRL-YOLO network structure that is both micro and fast.

**Figure 1:**
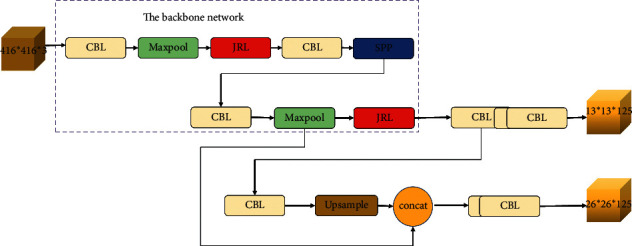
Structure of the proposed jump-join repetitious learning-YOLO network.

**Figure 2:**
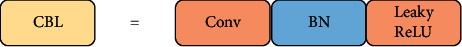
Structure of the convolution-batch normalization-leaky ReLU layer.

**Figure 3:**
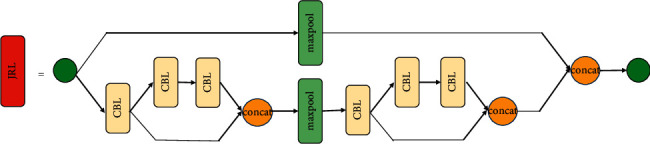
Structure of jump-join repetitious learning.

Figure 1 shows the overall network structure we proposed. The input image is set as 416 × 416, and the output feature sizes of the detection layer are 13 × 13 and 26 × 26, respectively. As shown in Figure 2, the convolution-batch normalization-leaky ReLU (CBL) layer represents a layer that includes the convolution layer, the layer normalization, and the use of leakey ReLU as the activation function. Maxpool indicates that this layer is the largest pooling layer, and upsample indicates that this layer is the upper sampling layer, with SPP being the spatial pyramid pooling structure.  Figure 3 shows the proposed JRL structure, which can effectively extract the features of targets. The JRL and SPP structures are used as the backbone network of YOLOv3-Tiny.

### 3.2. The Jump-Join Repetitious Learning Structure

The backbone network, as the feature extractor of the deep neural network, plays a very important role in the follow-up target detection. The performance of the backbone network is directly related to the speed and accuracy of target detection and also to the real-time performance and accuracy of the whole model. As the number of layers of the backbone network increases, the feature map extracted from the backbone network usually becomes smaller, which brings a certain amount of difficulty in recognition. Therefore, a more refined backbone network structure is needed to effectively extract the deep-level feature information of the target to enhance the accuracy of target recognition.

In this part, a JRL structure is proposed and used as part of the YOLOv3-Tiny backbone network to improve the target detection accuracy. The main advantage of JRL is that the multiscale features of different network levels can be connected by jumping, and the gradient semantic information of different levels can be repeatedly learned in the backpropagation of the network so that the whole network can achieve high accuracy with few parameters. This will be demonstrated in Section 4.

**Figure 4:**
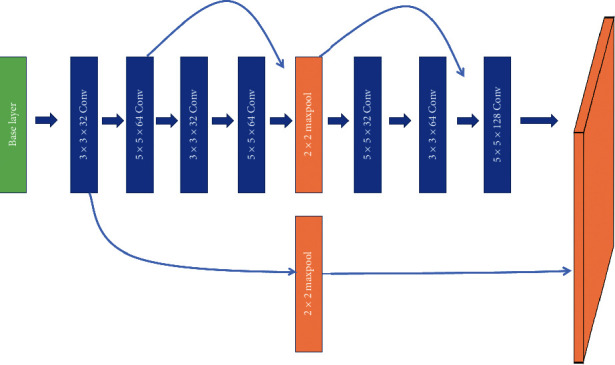
Convolution structure of jump-join repetitious learning.

As shown in Figure 4, convolution structures with convolution kernels of 3 × 3 and 5 × 5 are used in this structure. After the convolution operation of the 5 × 5 convolution kernel, a 3 × 3 convolution operation is carried out for the depth features, and then the features obtained from the first 5 × 5 convolution are concatenated after the 5 × 5 convolution.

The 5 × 5 convolution kernel can enhance the size of the receptive field of the image, and the larger receptive field information obtained by the 3 × 3 convolution check can be enhanced. Then, the depth features obtained by the 5 × 5 convolution can be combined with the features obtained by the first 5 × 5 convolution, which can enrich the semantic information of the network layer. The network layer with rich semantic information continues to propagate forward to the full connection layer. Compared with the CNN full connection layer which only has the convolutional layer and pooling layer, it can enhance the efficiency of feature extraction for finite pixels. Target features are more discriminable before classification, and the proposed structure can improve the detection accuracy and reduce misidentification compared to the CNN.

As shown in Figure 4, the base layer is *x*_0_, and ^*∗*^ is the presentation of the convolution. The backpropagation is used to update the weight, where the function is *f*, and *g*_*i*_ is used to represent the gradient propagated to the *i*th layer, so the updated equation can be expressed as

(1) $$\begin{array}{c} w_{1}^{\prime }=f\left(w_{1},g_{0}\right),\\ w_{5}^{\prime }=f\left(w_{4},g_{4},g_{2}\right),\\ w_{10}^{\prime }=f\left(w_{9},g_{7},g_{5},g_{0}\right). \end{array}$$

We can see that, in (1), the gradient information *g*_2_, *g*_0_, *g*_5_, and *g*_7_ in *w*_5_′ and *w*_10_′ is repeatedly used to update different weights. This indicates that different convolutional layers are repeatedly learning the corresponding gradient information, which not only increases the learning ability of the CNN but also simplifies the network structure.

### 3.3. Spatial Pyramid Pooling

In the current research, the detection of dangerous targets and dangerous actions usually faces the following challenges: first, the small targets that are dangerous items occupy few pixels, which brings difficulties to feature extraction. Second, with the movement of the car, the geometric proportion of the same dangerous item or dangerous action in the image usually changes, so the features extracted from the same object at different times may vary, which brings difficulties to the final classification and recognition. In addition, images in real-life situations are often affected by shadows, light, and other external factors. Therefore, we introduce the SPP structure into the backbone network of YOLOV3-Tiny [28].

**Figure 5:**
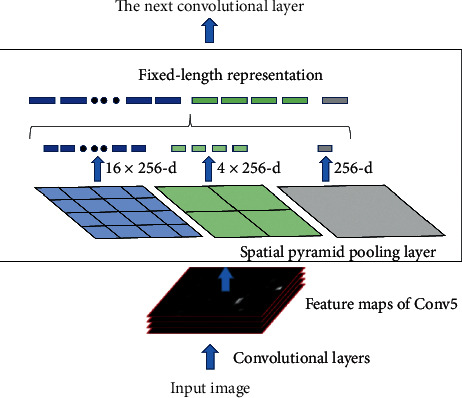
Structure of spatial pyramid pooling.

The SPP structure uses multisize pooling for the same set of depth features, which is equivalent to using different levels of semantic information. Finally, concating can produce fixed-sized outputs from images with inputs of any sizes. As shown in Figure 5, depth features can be obtained by splicing asynchronously long pooling layers, which can improve the scale invariance of images with different aspect ratios and sizes and reduce overfitting.

The SPP structure is placed after the first JRL structure as a further representation of the depth features of small targets can prevent the overfitting phenomenon caused by excessive numbers of convolutional layers in subsequent networks. The output of the SPP structure is connected to the second JRL structure. The addition of the SPP structure can effectively improve the detection accuracy of targets of different sizes.

### 3.4. Soft-NMS considering the Center Distance

Usually, in target detection, the model predicts multiple bounding boxes. The NMS algorithm is used to suppress redundant bounding boxes in postprocessing. The NMS process in YOLOv3-Tiny is described as follows:

  Step 1: set up the initial set of detection boxes *B*={*b*_1_, ..., *b*_*N*_}
  Step 2: set up the corresponding set of detection scores *S*={*s*_1_, ..., *s*_*N*_}
  Step 3: set up the final result set *D*={*d*_1_, ..., *d*_*N*_}
  Step 4: select the bounding box with the highest score from set *B*, denoted by *M*
  Step 5: add *M* to set *D*, and remove *M* from set *B*
  Step  6: calculate the IOU values of the remaining bounding boxes in *B* and *M*
  Step 7: delete all boxes in set *B* whose intersection ratio is greater than the overlap threshold *N*_*t*_
  Step 8: repeat steps 4 to 7 until set *B* is empty

The NMS formula of YOLOv3-Tiny is expressed as

(2) $$\begin{array}{c} s_{i}=\left\{\begin{array}{c} s_{i},\text{ iou}\left(M,b_{i}\right)<N_{t}\\ 0,\text{ iou}\left(M,b_{i}\right)\geq N_{t} \end{array}\right., \end{array}$$

where *s*_*i*_ represents the score of bounding boxes, *M* represents the highest score of all generated bounding boxes, and iou(*M*, *b*_*i*_) represents the IOU of bounding boxes *b*_*i*_ and *M*. When the iou(*M*, *b*_*i*_) is greater than or equal to the overlap threshold *N*_*t*_ set by configuring the superparameter, the score of bounding boxes *i* will be directly set to 0, which is equivalent to direct deletion.

The soft-NMS algorithm is an NMS algorithm for accurate target detection and location proposed by Bodla et al. [29]. Compared with the NMS algorithm, soft-NMS not only draws more accurate suppression boxes for detected targets but also improves the average accuracy using public data. Soft-NMS can be expressed as

(3) $$\begin{array}{c} s_{i}=\left\{\begin{array}{c} s_{i},\text{ iou}\left(M,b_{i}\right)<N_{t}\\ s_{i}e^{\left(-\left(\text{iou}{\left(M,b_{i}\right)}^{2}\right)/\sigma \right)},\text{ iou}\left(M,b_{i}\right)\geq N_{t} \end{array}\right., \end{array}$$

where *s*_*i*_ represents the score of bounding boxes, *M* presents the highest score of all generated bounding boxes, iou(*M*, *b*_*i*_) represents the IOU of bounding boxes *b*_*i*_ and *M*, and *σ* is the superparameter. When iou(*M*, *b*_*i*_) is greater than or equal to the overlap threshold *N*_*t*_, the core of soft-NMS is to add penalty terms to reduce its confidence, and its score will be reduced accordingly.

**Figure 6:**
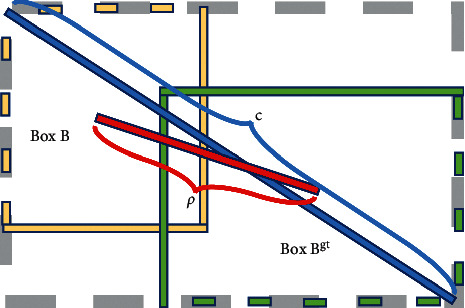
Center distance model of the detection box.

In this paper, inspired by DIoU-NMS [30], we introduce the center distance ratio (CDR) of two detection boxes on the basis of the Gaussian weighted soft-NMS. The formula of CDR is as follows:

(4) $$\begin{array}{c} R=\frac{\rho^{2}\left(b,b^{gt}\right)}{c^{2}}, \end{array}$$

where *b* and *b*^*gt*^ are the center points of the box *B* and *B*^*gt*^, separately, and *ρ* is the Euclidean distance, with *c*^2^ representing the diagonal distance of the minimum bounding box *B*and *B*^*gt*^. As shown in Figure 6, the square of the ratio between the center distance of the two detection boxes and the diagonal distance of the minimum surrounding box is used as the measurement *R* in order to measure the distance between two bounding boxes.

After introducing the CDR, the NMS algorithm in (3) can be improved, which is expressed as

(5) $$\begin{array}{c} s_{i}=\left\{\begin{array}{c} s_{i},\text{ iou}\left(M,b_{i}\right)-R<\varepsilon \\ s_{i}e^{-\left(\left(\text{iou}{\left(M,b_{i}\right)}^{2}\right)/\sigma \right)},\text{ iou}\left(M,b_{i}\right)-R\geq \varepsilon \end{array}\right., \end{array}$$

where *s*_*i*_ is the score of the bounding box and *M* represents the maximum score among all generated bounding boxes. iou(*M*, *b*_*i*_) is the IOU of the bounding box *b*_*i*_ and *M*, and *σ* is the superparameter. When the difference value between iou(*M*, *b*_*i*_) and *R* is greater than or equal to the overlap threshold *N*_*t*_, the penalty terms will be increased to reduce its confidence, and its score will decrease accordingly.

The improved NMS algorithm process in JRL-YOLO can be implemented as follows:

  Step 1: set up the initial set of detection boxes *B*={*b*_1_, ..., *b*_*N*_}
  Step 2: set up the corresponding set of detection scores *S*={*s*_1_, ..., *s*_*N*_}
  Step 3: set up the final result set *D*={*d*_1_, ..., *d*_*N*_}
  Step 4: select the bounding box with the highest score from set *B*, denoted by *M*
  Step 5: add *M*to set *D*, and remove *M* from set *B*
  Step  6: calculate the IOU values of the remaining bounding boxes in set *B* with *M*
  Step 7: calculate *c*^2^, the diagonal distance of the minimum bounding box in set *B* with *M*
  Step 8: calculate *ρ*^2^(*b*, *b*^*gt*^), the Euclidean distance of the bounding box in set *B* with *M*
  Step 9: calculate *R*, where *R*=((*ρ*^2^(*b*, *b*^*gt*^))/*c*^2^)
  Step 10: calculate *N*_*t*_′=iou(*M*, *b*_*i*_) − *R*
  Step 11: calculate *s*_*i*_=*s*_*i*_*e*^−(iou(*M*, *b*_*i*_)^2^/*σ*)^ in set *B* whose *N*_*t*_′ is greater than the overlap threshold *ε*
  Step 12: if the value of *s*_*i*_ is greater than the IOU threshold displayed by the setting, it is reserved
  Step 13: repeat steps 4 to 12 until set *B* is empty

When the IOU value of the two boxes is relatively large and the distance between the centers of the two boxes is relatively large, too, the two boxes are objects that are close to each other. This reduces the possibility of two close objects being misjudged as the same object detection box. The following experimental section shows that the phenomenon of missing detection is reduced effectively, and the evaluation result evidences that the proposed algorithm is more practical than state-of-the-art approaches.

## 4. Experiments' Results and Analysis

In this section, the performance of the proposed JRL-YOLO network is evaluated. This section mainly introduces the experimental equipment and parameter setting, the collection of experimental data, and the comparison of experimental results. A large number of experiments were carried out on the self-made dataset and VOC2007 [31] to verify the performance of the proposed model, which was transplanted to an embedded development board to test the speed of running.

### 4.1. Experimental Platform and Parameter Setting

The hardware and software configuration platform of the experiments are shown in Table 2, in which the NVIDIA Jetson AGX Xavier board will be embedded into a car for outdoor experiments. To ensure the fairness of the experimental results, all initial algorithm parameters of the experiments are maintained the same, which are given as follows: width = 416, height = 416, learning_rate = 0.001, batch = 64, subdivisions = 8, steps = (16,000, 20,000), and max_batches = 30,000.

All models were trained on an NVIDIA GeForce RTX 2070 GPU. The data training in the experiments used the open-source Darknet framework provided by the AlexeyAB team, available in [32]. The version of CUDA is 10.0, and the version of cuDNN is 7.6. In order to verify the speed of the model on embedded system devices, the model was later transplanted to NVIDIA Jetson AGX Xavier to test the speed. NVIDIA Jetson AGX Xavier also uses the Darknet framework, and the operating system is Ubuntu 18.04. The versions of CUDA and cuDNN are also 10.0 and 7.6, respectively.

### 4.2. Details of Experimental Data Collection

We analyzed several common types of targets that may appear in the campus, dividing the dangerous targets into two categories when collecting and making the datasets, dangerous objects and dangerous actions. Dangerous objects mainly include the gun, knife, stick, and short knife, and dangerous actions include fight, kick, and fall. The dataset has overall 6809 pictures, among which each class occupies a relatively balanced proportion of the total dataset. In this dangerous target dataset, we randomly selected 80% of the data for training and the remaining 20% for testing. In fact, there is some uncertainty in such data selection [33], so we also use open datasets for validation, and explicit consideration of these issues may provide better knowledge of the authenticity of the results.

**Figure 7:**
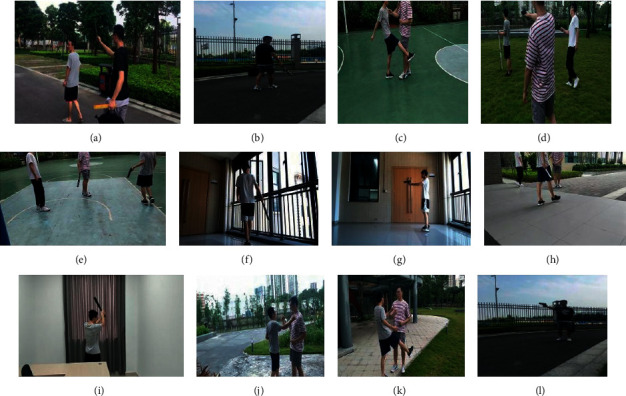
Self-made dangerous target datasets in different outdoor scenarios.

The dataset produced in this study includes images from the internet and images taken by ourselves. The data collection follows the following rules: (1) two methods of manual shooting and vehicle collection are used; (2) discrete acquisition of images in the same video is applied, that is, an image is collected at a fixed time interval; (3) dangerous objects and dangerous actions are sampled in multiple outdoor scenes.  Figure 7 shows examples of the collected dangerous dataset.

In order to verify the robustness and universality of our model, the model was validated on the public dataset Pascal VOC2007. Pascal VOC2007 is an open dataset that is often used for image classification, target detection, and target segmentation. The VOC2007 dataset consists of 20 main classes of 9963 images, of which 5011 are used for training and 4952 are used for testing. Among the 20 categories, the number of pictures of people is relatively large, whereas the number of other categories is relatively small. Thus, there is a phenomenon of data imbalance. However, this makes the performance of our model more prominent on the Pascal VOC2007 dataset.

### 4.3. Comparison of Experimental Results

#### 4.3.1. Comparison of Precision

In this test, 1358 pictures of 7 kinds of objects were randomly selected from our self-made dataset of dangerous objects in each scene, with each kind consisting of 194 pictures. The seven specific categories are named kick, hit, fall, gun, knife, stick, and short knife. The accuracy of JRL-YOLO was evaluated by cross-validation. Six clustering centers of JRL-YOLO were used with sizes of (10,14), (23,27), (37,58), (81,82), (135,169), and (344,319). In the VOC2007 dataset, a common test set containing 4952 images was used for testing.

The exact category and prediction category of samples can be divided into four categories [34]: TP (true positive), FP (false positive), TN (true negative), and FN (false negative), which are defined as follows:

- TP: a positive sample predicted by the model as a positive class
- TN: a negative sample predicted by the model as a negative class
- FP: a negative sample predicted by the model as a positive class
- FN: a positive sample predicted by the model as a negative class

**Figure 8:**
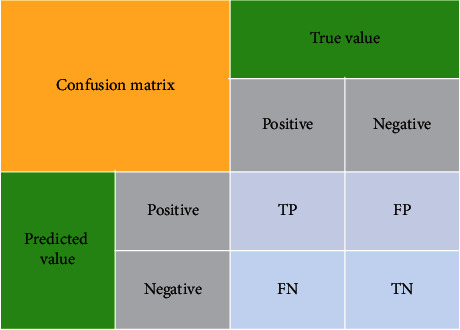
Confusion matrix: schematic diagram of TP, FP, FN, and TN.

Let us see Figure 8 to better understand the meaning of TP, TN, FP, and FN. The “true value” represents the true value of an object, and the “predicted value” is the predicted value obtained by JRL-YOLO after calculation. TP, FP, FN, and TN are the cases where positive values are predicted correctly, negative values are predicted falsely, truth values are predicted falsely, and negative values are predicted correctly, respectively.

Precision refers to the proportion occupied by TP in all positive classes judged by the model (TP + FP). High precision means that most of the results detected by the model are correct. The recall rate is the proportion of TP correctly judged by the model in all positive examples (TP + FN) in the dataset. The higher the recall rate is, the more objects the model can find in the picture. Generally, in order to evaluate the accuracy between different types of targets, MAP [35] is one of the important measures to evaluate test results. The precision and recall rate are defined as follows:

(6) $$\begin{array}{c} \text{precision}=\frac{\text{TP}}{\text{TP}+\text{FP}},\\ \text{recall}=\frac{\text{TP}}{\text{TP}+\text{FN}}. \end{array}$$

The values of TP, FP, FN, and TN are judged according to the values of IOU. When we calculate the values of precision and recall, we can draw the PR curve to get the values of AP.

In order to verify the superiority of the JRL-YOLO model in the lightweight sense, YOLOv2-Tiny and YOLOv3-Tiny were trained and tested on the self-made dangerous target dataset. The statistical comparison experiments of the three groups using AP and MAP as measurements are shown in Table 3. According to Postorino and Versaci [36], considering the uncertainty of experimental data results in very few cases during the experimental process, the potential aggregation of objects in the identification process is not as absolutely clear as it is indicated. For example, there are very few misidentification phenomena between sticks and knives. In order to ensure the fairness of the experiments, the confidence of the object category in all the experiments in this paper is set to 0.6. This value can also be adjusted to achieve higher accuracy.

As obviously revealed in Table 3, regardless of whether complex targets are included, the AP and MAP of each target in the JRL-YOLO network are significantly higher than those of YOLOv2-Tiny and YOLOv3-Tiny. By replacing the backbone network of YOLOv3-Tiny with the combination of JRL and SPP, the MAP value of the proposed JRL-YOLO network model is increased by 11.74 percent compared with that of YOLOv2-Tiny and by 3.22 percent compared with that of YOLOv3-Tiny.

An important advantage of JRL-YOLO is that it can quickly and efficiently extract deep features of objects of different sizes and overcome the problem of low accuracy of target recognition of any size and proportion. In addition, the introduction of the SPP structure can increase the size of the receptive field of the target, effectively overcoming the phenomenon of missing detection and misidentification caused by different geometric deformations of the same object. It also reduces the overfitting of the network. Due to the superior performance of the backbone network, the MAP of JRL-YOLO is 3.94% higher than that of YOLOv3-Tiny and 16.02% higher than that of YOLOv2-Tiny.

In the VOC2007 dataset, a total of four groups of comparative experiments were selected. The comparison of AP and MAP results is shown in Table 4. It can be seen that only the MAP values of bicycle, car, chair, cow, sheep, and TV monitor in JRL-YOLO are lower than those of YOLOv4-Tiny, whereas the MAP values of other classes are the highest. This may be due to the JRL-YOLO's smaller number of model parameters. Moreover, the MAP of JRL-YOLO is 23.47% higher than that of YOLOv3-Tiny and 5.12% higher than that of YOLOv4-Tiny. Therefore, it can be concluded that the proposed JRL-YOLO network has high universality and is suitable for the detection of most types of targets.

#### 4.3.2. Comparison of Speed and Model Sizes

Four groups of experiments were selected to compare the model size and MAP on the self-made dangerous target dataset and VOC2007, respectively. As shown in Table 5, it is obvious that, on the self-made dangerous target dataset, the size of the JRL-YOLO network model is only 5.84 MB, which is nearly 6 times smaller than that of the YOLOv3-Tiny model and 8 times smaller than that of the YOLOv2-Tiny model, while showing the highest accuracy. Similarly, on the VOC2007 dataset, JRL-YOLO has the highest accuracy but the lowest model size.

In order to verify the real-time performance of JRL-YOLO, input videos of different sizes were used to test the proposed JRL-YOLO on NVIDIA GeForce RTX 2070 and NVIDIA Jetson AGX Xavier. As shown in Table 6, with the size of the input video becoming smaller, the frame rate detected increased. The average FPS detection on NVIDIA GeForce RTX 2070 was 98.4 with a video of 1080 × 1920 frame size and 150.0 with a video of 640 × 480 frame size. Thus, the smaller the frame size of the video is, the larger the FPS will be. Real-time detection can also be achieved on NVIDIA Jetson AGX Xavier for videos with frame sizes being up to 1280 × 720.

The above results show that the JRL-YOLO model is lightweight, highly precise, and performing in real time.

#### 4.3.3. Qualitative Analysis

**Figure 9:**
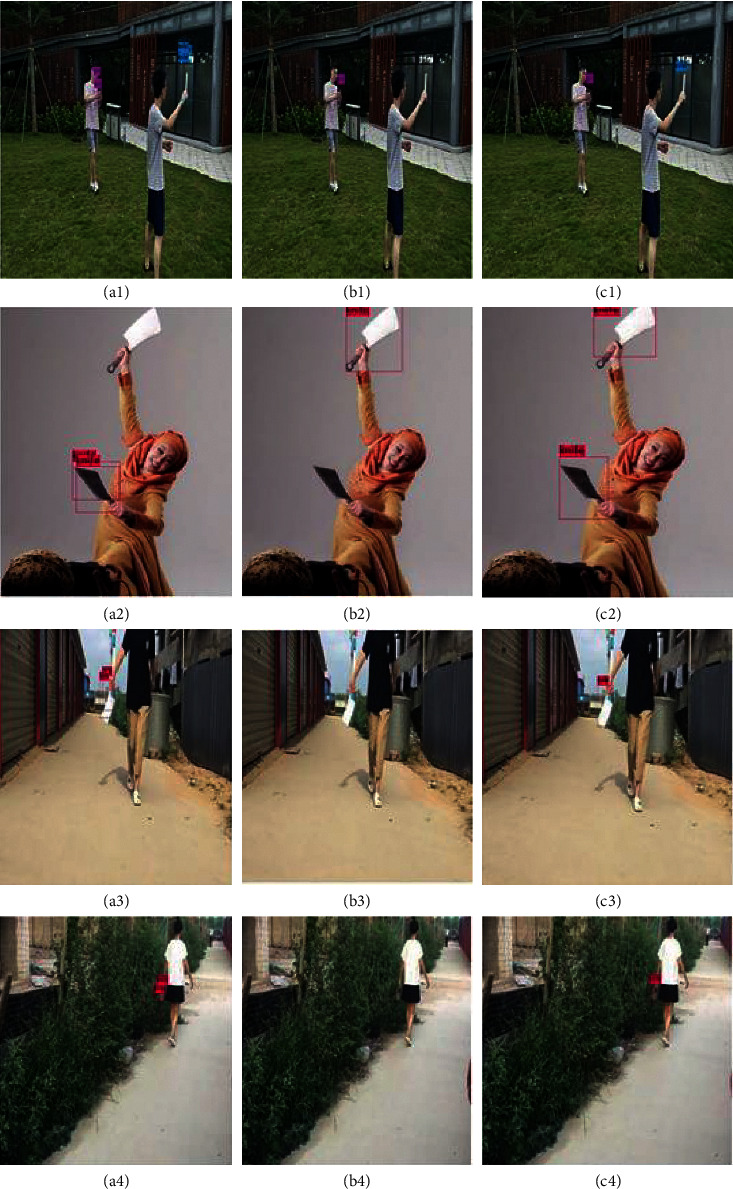
Comparison results of YOLOv2-Tiny (a1–a4), YOLOv3-Tiny (b1–b4), and the proposed JRL-YOLO (c1–c4) tested on the dangerous target dataset.

In Figure 9, we selected 12 detection results of 4 pictures from the test set of our self-made dangerous target dataset to prove the superiority of the JRL-YOLO algorithm. The pictures in the first column are the detection results of YOLOv2-Tiny, the pictures in the second column are the detection results of YOLOv3-Tiny, and the pictures in the third column are the detection results of our proposed JRL-YOLO. It can be clearly seen that target detection is missing in the YOLOv3-Tiny results in Figure 9b(1)–b(4). Although the detection of YOLOv2-Tiny is better than that of YOLOv3-Tiny, the detection results have overlapping frames in Figure 9a(1)–a(4). Additionally, our proposed method can detect small targets, and the detected box is closer to the target in Figure 9c(1)–c(4). The four groups of experiments show that, by improving the backbone network of feature extraction and adding the SPP structure, the proposed JRL-YOLO improves the performance of target detection in real application scenarios.

The original image of second line in Figure 8 is available online at http://www.sanqin.com/2016/0415/200756.shtml.

**Figure 10:**
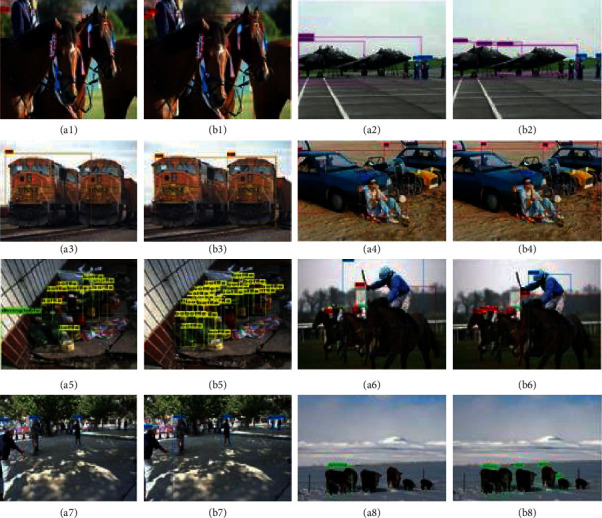
Comparison results of YOLOv3-Tiny (a1–a8) and the proposed JRL-YOLO (b1–b8) tested on VOC2007.

In order to prove that JRL-YOLO also has superior performance in other types of target detection, eight images were selected from the VOC2007 dataset, tested with utilizing YOLOv3-Tiny and JRL-YOLO, respectively. The test results are shown in Figure 10. a(1)–a(8) which are the detection results of YOLOv3-Tiny, and b(1)–b(8) are the detection results of JRL-YOLO. By comparison, it is obvious that YOLOv3-Tiny has missed detection in every picture, while the detection result of JRL-YOLO is much better than that of YOLOv3-Tiny. In b(7) and b(8), JRL-YOLO also has a missing detection phenomenon, which may be caused by too few parameters. Thus, it can be seen that JRL-YOLO has better feature extraction abilities and leads to higher detection accuracy than that of YOLOv3-Tiny.

#### 4.3.4. Comparison of Loss Curves

**Figure 11:**
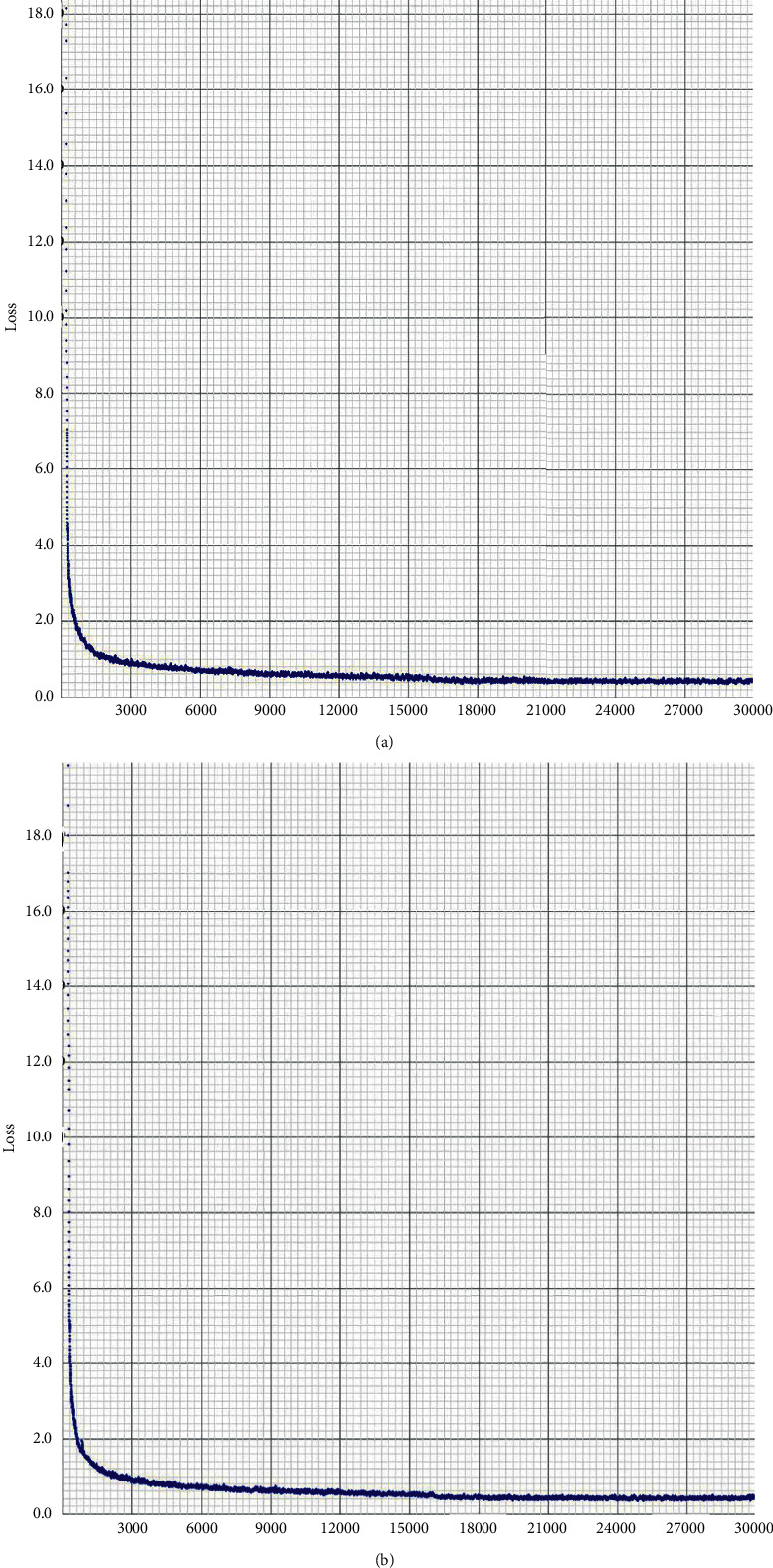
Comparison of loss curves for two networks: (a) YOLOv3-Tiny network; (b) JRL-YOLO network.

As shown in Figure 11, the final avg_loss calculated by JRL-YOLO in Figure 11(b) is 0.4544, while avg_loss of YOLOv3-Tiny is 0.4693. The result of JRL-YOLO is 0.0149 smaller than avg_loss of YOLOv3-Tiny. The convergence trend of both is basically the same, but avg_loss of the JRL-YOLO model is smaller, which indicates that the JRL-YOLO model has better convergence.

#### 4.3.5. Comparison of the Improved NMS

**Figure 12:**
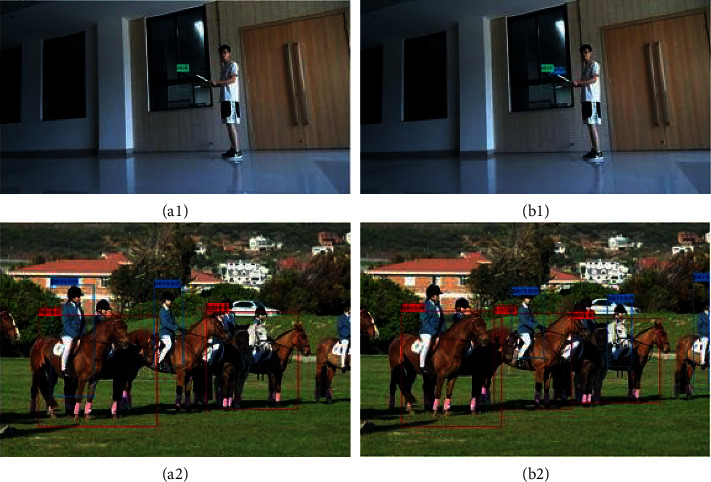
Comparison results of JRL-YOLO using NMS (a1, a2) and JRL-YOLO using the improved NMS (b1, b2).

Figure 12 shows the comparison of two NMS algorithms tested on dangerous targets employing the proposed JRL-YOLO. Obviously, we can see that when two targets are close to each other, two bounding boxes inhibit each other, resulting in one of the detection boxes being inhibited, and the detection missing phenomenon appears in Picture (a1) and Picture (a2). As for the improved NMS algorithm, it effectively reduces the missed detection phenomenon in Picture (b1) and Picture (b2). Thus, the improved NMS algorithm has greatly improved the detection accuracy of the targets.

## 5. Conclusions

This paper proposes an improved YOLOv3-Tiny real-time detection algorithm for dangerous targets on campus. The improved microtarget detection model is called jump-join repetitious learning YOLO. The main improvement of the JRL-YOLO network lies in a new backbone network composed of the JRL structure and SPP structure. In addition, the proposed method also improves the NMS algorithm by adding the center distance measurement of the two object detection boxes to the soft-NMS algorithm, so as to have high detection accuracy when detecting two objects with a close distance. By extracting multiscale features of images and increasing the ability of repeated learning of the network, the new network structure can improve the accuracy of the original algorithm while reducing the size of the model. To a certain extent, the computational burden is reduced, and the accuracy of target detection is improved. The new network structure is also easy to be transplanted to other algorithm models.

Compared with the original YOLOv3-Tiny model, JRL-YOLO designed in this paper has a smaller volume and higher precision. Meanwhile, it can achieve real-time detection on the embedded system NVIDIA Jetson AGX Xavier. The experimental results show that JRL-YOLO has the advantages of few model parameters, high accuracy, and fast detection speed. It can complete the real-time detection task of dangerous targets on campus in complex outdoor scenes, and JRL-YOLO greatly reduces the missing detection phenomenon in the detection process when using YOLOv3-Tiny.

In the future work, the polynomial convolution kernel will be used to continue to improve the feature extraction capability of the backbone network, so as to improve the efficiency of feature extraction and the performance of detection. The detection of small targets usually depends on larger network structures, and the future work will also include small-target detection in lightweight networks and building a more efficient feature matching mechanism in the detection layer. The method in this paper can also provide references for abnormal behavior detection in the field of security.

## Figures and Tables

**Table 1:** The YOLOv3-Tiny network structure.

Layer	Type	Filters	Size/stride	Output
0	Convolutional	16	3 × 3/1	416 × 416 × 16
1	Maxpool	—	2 × 2/2	208 × 208 × 16
2	Convolutional	32	3 × 3/1	208 × 208 × 32
3	Maxpool	—	2 × 2/2	104 × 104 × 32
4	Convolutional	64	3 × 3/1	104 × 104 × 64
5	Maxpool	—	2 × 2/2	52 × 52 × 64
6	Convolutional	128	3 × 3/1	52 × 52 × 128
7	Maxpool	—	2 × 2/2	26 × 26 × 128
8	Convolutional	256	3 × 3/1	26 × 26 × 256
9	Maxpool	—	2 × 2/2	13 × 13 × 256
10	Convolutional	512	3 × 3/1	13 × 13 × 512
11	Maxpool	—	2 × 2/1	13 × 13 × 512
12	Convolutional	1024	3 × 3/1	13 × 13 × 1024
13	Convolutional	256	1 × 1/1	13 × 13 × 256
14	Convolutional	512	3 × 3/1	13 × 13 × 512
15	Convolutional	36	1 × 1/1	13 × 13 × 36
16	YOLO	—	—	—
17	Route 13	—	—	—
18	Convolutional	128	1 × 1/1	13 × 13 × 128
19	Upsample	—	2 × 2/1	26 × 26 × 128
20	Route 19, 8	—	—	—
21	Convolutional	256	3 × 3/1	26 × 26 × 256
22	Convolutional	36	1 × 1/1	26 × 26 × 256
23	YOLO	—	—	—

**Table 2:** Software and hardware configurations of the experimental platform.

Software and hardware	NVIDIA GeForce RTX 2070	NVIDIA Jetson AGX Xavier
Operating platform	Windows 10	Ubuntu 18.04
CPU/GHz	i7-9750H,G5 5590, CPU@2.60 GHz,2.59 GHz	8 nuclear ARM 64 8 MB L2 + 4 MB L3
GPU	CUDA 10.0, cuDNN 7.6	CUDA 10.0, cuDNN 7.6
RAM	16 GB	16 GB
Deep learning framework	Darknet	Darknet

**Table 3:** Comparison of using three different lightweight models on the dangerous target dataset.

Precision	Classes	YOLOv2-Tiny	YOLOv3-Tiny	JRL-YOLO
AP (%)	Gun	75.58	85.92	89.26
	Knife	38.76	71.66	80.50
	Hit	70.71	81.64	81.77
	Kick	91.68	88.36	94.85
	Fall	85.01	87.20	88.08
	Stick	74.03	81.58	88.18
	Short knife	75.14	77.66	81.25

MAP (%)	—	73.29	81.81	85.03

**Table 4:** Comparison of MAP results tested on the VOC2007 dataset.

Precision	Classes	YOLOv2-Tiny	YOLOv3-Tiny	YOLOv4-Tiny	JRL-YOLO
AP (%)	Aeroplane	39.83	46.47	54.06	**59.63**
	Bicycle	53.10	54.24	**62.13**	59.90
	Bird	13.55	18.83	29.55	**33.03**
	Boat	15.83	18.31	26.73	**31.49**
	Bottle	3.82	8.79	16.40	**18.56**
	Bus	45.52	46.45	57.06	**62.29**
	Car	52.53	60.62	**72.81**	71.21
	Cat	35.10	35.77	50.12	**53.93**
	Chair	16.51	21.12	**31.11**	28.66

MAP (%)	Cow	29.16	31.62	**47.76**	42.56
	Dining table	27.37	28.42	35.47	**44.62**
	Dog	28.53	30.11	41.00	**46.74**
	Horse	55.40	57.84	65.57	**67.99**
	Motorbike	52.18	54.37	65.30	**65.52**
	Person	39.47	48.79	63.73	**64.41**
	Potted plant	6.84	10.87	18.77	**21.31**
	Sheep	31.36	40.85	**46.70**	42.97
	Sofa	21.15	25.72	36.54	**44.79**
	Train	46.75	51.56	59.32	**68.24**
	TV monitor	36.45	42.21	**48.06**	46.91
	—	35.43	39.59	46.50	**48.88**

The best result is shown in bold.

**Table 5:** Average MAP of two datasets and comparison of their model sizes.

		YOLOv2-Tiny	YOLOv3-Tiny	YOLOv4-Tiny	JRL-YOLO
Dangerous target dataset	Model size (MB)	42.1	33.1	22.4	**5.84**
	MAP (%)	73.29	81.81	84.49	**85.03**

Pascal VOC2007 dataset	Model size (MB)	42.2	33.2	22.5	**6.58**
	MAP (%)	35.43	39.59	46.50	**48.88**

The best result is shown in bold.

**Table 6:** FPS comparison of input videos of different sizes.

Video frame size	NVIDIA GeForce RTX 2070 (FPS)	NVIDIA Jetson AGX Xavier (FPS)
1080 × 1920	98.4	23
1280 × 720	108.5	25
640 × 480	150.0	34

## Data Availability

The labeled datasets used to support the findings of this study are available from the corresponding author upon request.
